# Immune-Checkpoint Induced Skin Toxicity Masked as Squamous Cell Carcinoma: Case Report on Mimickers of Dermatological Toxicity with PD-1 Inhibition

**DOI:** 10.3390/curroncol30050342

**Published:** 2023-04-27

**Authors:** Sze Wah Samuel Chan, Rahul Shukla, Jennifer Ramsay, Elaine McWhirter, Paul Barnfield, Rosalyn A. Juergens

**Affiliations:** 1 Department of Oncology, McMaster University, Hamilton, ON L8V 5C2, Canada; 2 Division of Medical Oncology, Juravinski Cancer Center, Hamilton, ON L8V 5C2, Canada; 3 Faculty of Health Sciences, McMaster University, Hamilton, ON L8S 4L8, Canada; 4 Department of Pathology and Molecular Medicine, McMaster University, Hamilton, ON L8S 4L8, Canada

**Keywords:** anti-PD-1 inhibitor, immune-related adverse events, cutaneous immune-related adverse events, squamous cell carcinoma, immune checkpoint inhibitor, pembrolizumab

## Abstract

Background: Immune checkpoint inhibitors (ICI) are increasingly the mainstay of oncology treatment. Immune-related adverse events (irAEs) from ICI therapy differ from cytotoxic adverse events. Cutaneous irAEs are one of the most common irAEs and require careful attention to optimize the quality of life for oncology patients. Patient and Methods: These are two cases of patients with advanced solid-tumour malignancies treated with PD-1 inhibitor therapy. Results: Both patients developed multiple pruritic hyperkeratotic lesions, which were initially diagnosed as squamous cell carcinoma from skin biopsies. The presentation as squamous cell carcinoma was atypical and, upon further pathology review, the lesions were more in keeping with a lichenoid immune reaction stemming from the immune checkpoint blockade. With the use of oral or topical steroids and immunomodulators, the lesions resolved. Conclusions: These cases emphasize that patients on PD-1 inhibitor therapy who develop lesions resembling squamous cell carcinoma on initial pathology may require an additional pathology review to assess for immune-mediated reactions, allowing appropriate immunosuppressive therapy to be initiated.

## 1. Introduction

Immune checkpoint inhibitors (ICI) that block the programmed cell death protein 1 (PD-1)/programmed death-ligand 1 (PD-L1) axis or the cytotoxic T lymphocyte antigen-4 (CTLA-4)/CD28 axis are emerging as the standard of care for various advanced malignancies. Immune-related adverse events (irAEs) are toxicities with an immune origin related to off-target activation of the immune system that can manifest in various autoinflammatory conditions. Dermatological irAEs of any grade are one of the most common irAEs, occurring in about 40 to 50% of treated patients [1,2,3,4]. There are various manifestations including rash, pruritis, and vitiligo [4,5]. Furthermore, lichenoid reactions and lichen planus have also been reported in the literature [6,7]. Higher-grade adverse events can also occur such as bullous dermatoses [8], severe cutaneous adverse reactions (SCAR) such as Stevens–Johnson syndrome/toxic epidermal necrolysis [9], and drug reactions with eosinophilia and systemic symptoms (DRESS) or drug hypersensitivity syndrome (DHS) [10,11].

Skin irAEs remain poorly characterized and the spectrum of reactions remains to be fully elucidated. There are several case reports in the literature describing patients on ICIs who were initially diagnosed with squamous cell carcinomas, but further review revealed it was a lichenoid reaction due to PD-L1/PD-1 checkpoint inhibition [12,13,14,15]. Here, we report on two cases of ICI-induced dermatitis eruptions which were initially diagnosed with squamous cell carcinoma, but the clinical course and further pathological clarification were more consistent with a skin irAE. The purpose of this paper is to provide education to the medical oncology community about emerging skin irAEs and the initial steps in investigations and management.

## 2. Case Presentations

### 2.1. Case 1

A 74-year-old male initially presented with stage III lung adenocarcinoma of the right lower lobe with mediastinal node involvement. He was initially treated with curative intent with neoadjuvant chemotherapy and radiation using etoposide and cisplatin followed by surgical resection. Four years later, the cancer relapsed with intra- and extra-thoracic nodal disease. A re-biopsy for molecular evaluation was negative for driver mutations, but the tumour did have PD-L1 staining greater than 50% and he was treated with single-agent PD-1 inhibition with pembrolizumab.

His past medical history was remarkable for a history of squamous cell carcinoma of the left cheek and basal cell carcinoma of the left temple, which were both excised.

Approximately four months after starting pembrolizumab, the patient developed a bilateral widespread pruritic erythematous rash with some nodularity on the shins. Topical steroid cream therapy was initiated by the treating dermatologist. Skin biopsies of the nodular area were interpreted as moderate to well-differentiated keratinizing squamous cell carcinoma with keratoacanthomatous features. The patient was then referred to oncology for treatment of presumed multifocal squamous cell carcinoma on his lower legs. The patient was assessed by radiation oncology but given his clinical presentation of bilateral leg involvement with some response to topical steroid treatment, additional biopsies were requested to better target areas for potential radiation therapy. Multiple biopsies from each leg were taken, targeting and mapping the different nodular and erythematous areas.

**Figure 1:**
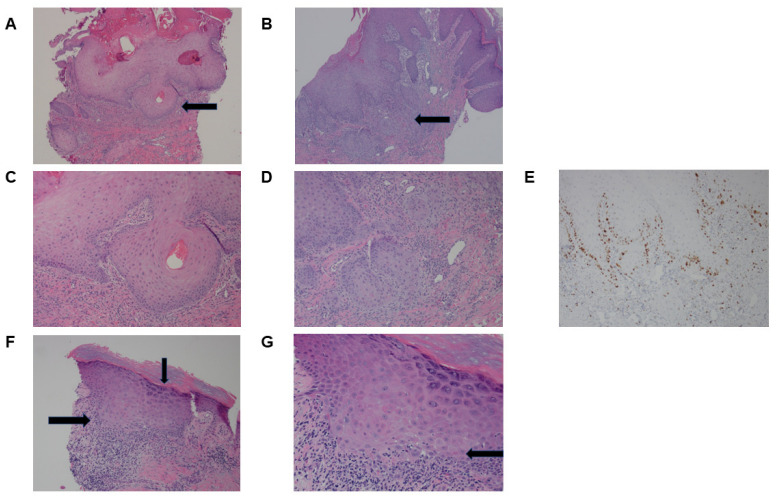
Case 1. Skin biopsies of the leg rash in Case 1. (**A**) An 88× magnification of the left leg biopsy of the patient’s rash showed psoriasiform acanthosis with an endophytic squamous proliferation (shown in the arrows). (**B**) An 88× magnification of the right leg biopsy which demonstrates more endophytic squamous proliferation compared to the left leg biopsy (highlighted by the arrows). Mild basal atypia with squamous maturation (**C**,**D**) and Ki-67 staining (**E**) indicates the proliferation was mainly limited to the basal layer. (**F**) A 220× magnification of the right leg biopsy which demonstrates the lichen planus inflammatory changes with band-like lymphohistiocytic infiltrates (left arrow), wedge-shaped hypergranulosis (vertical arrow), and overlying hyperkeratosis. (**G**) A 440× magnification of right leg biopsy with interface dermatitis obscuring the dermo–epidermal junction with occasional apoptosis (arrow).

The biopsies were subsequently referred for review by another pathologist associated with the oncology center. A review of the biopsies showed a psoriasiform acanthotic process with an endophytic squamous proliferation in the nodular areas and prominent lichenoid inflammatory changes in hypergranulosis, and some apoptosis in the erythematous areas (Figure 1A,B). The endophytic squamous proliferation was quite florid in some biopsies but showed no cytologic atypia; Ki67 proliferation confirmed proliferation limited to the basal layer (Figure 1C–E). These pathologic changes in conjunction with the clinical history were diagnosed as pseudoepitheliomatous (PEH) eruption of hypertrophic lichen planus (Figure 1F,G). There was no evidence of malignancy in any of the biopsies.

**Figure 2:**
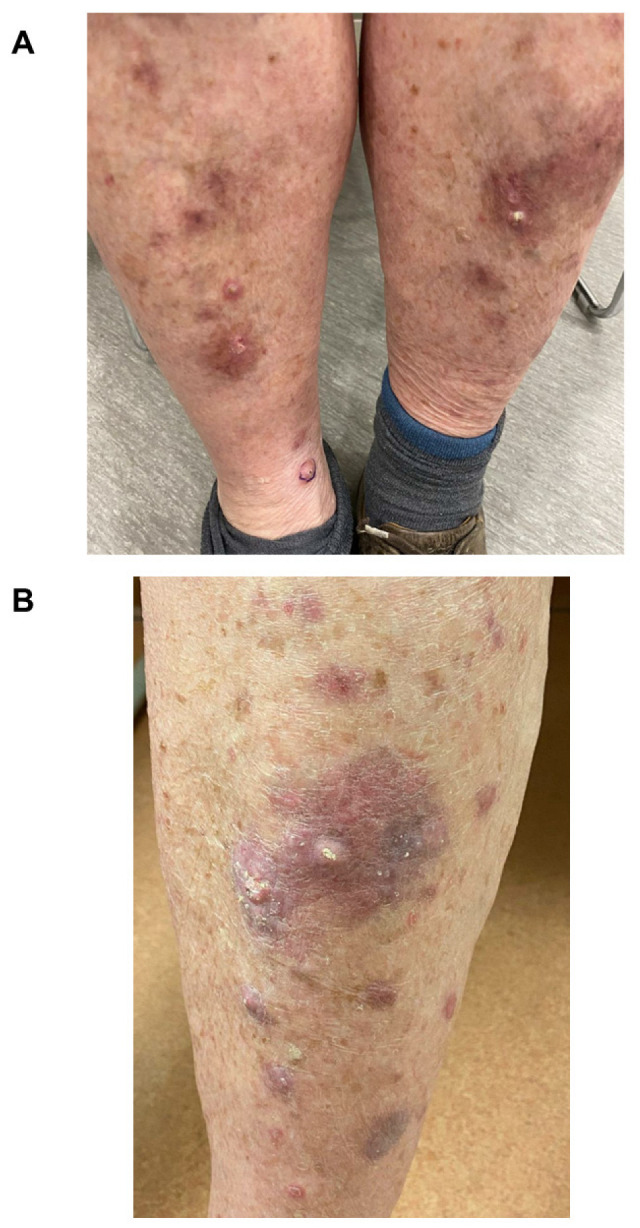
Case 1. Erythematous and nodular rash on the bilateral lower limbs before topical steroid treatment. (**A**) shows the bilateral lower leg violaceous papules before topical treatment. Biopsies of the right ankle lesion are shown in Figure 1B–G. (**B**) demonstrates a closer view of the left leg lesion, and the histopathological samples are shown in Figure 1A.

**Figure 3:**
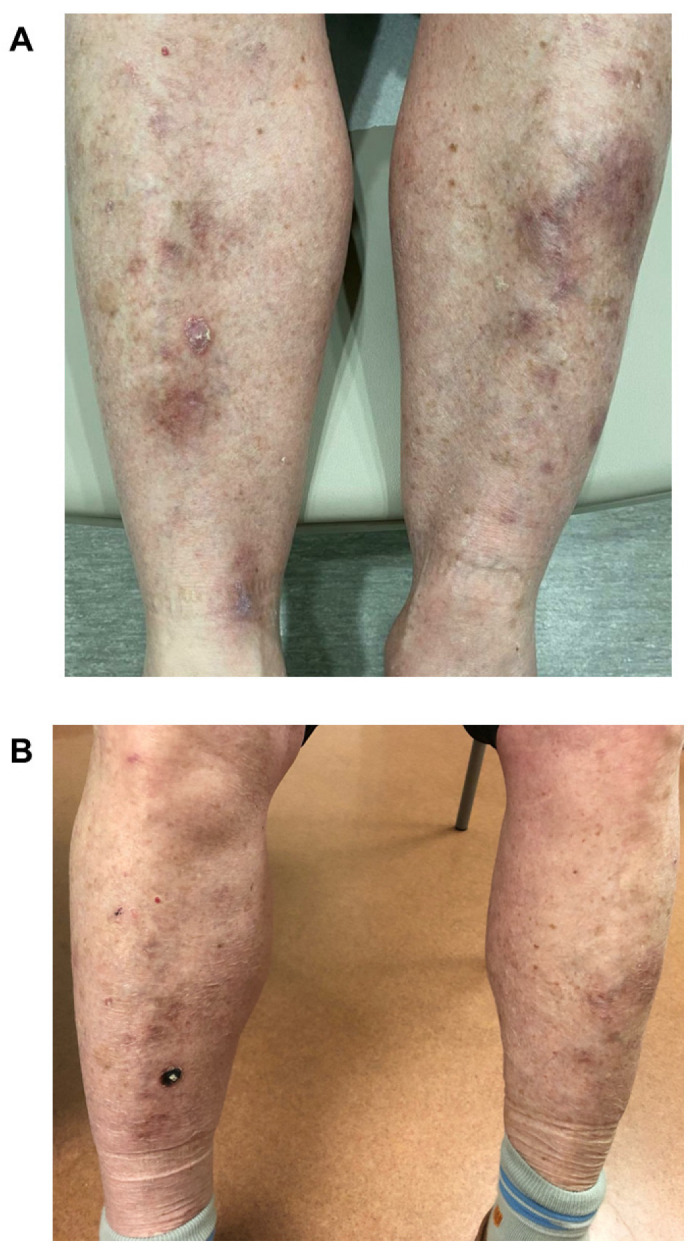
Case 1. Rash bilateral lower leg extremities. (**A**) Shows the improvement after 7 months of topical steroids and immunomodulators and (**B**) demonstrates the patient’s lesions remain under control a year after the initial rash appearance.

The patient’s rash on the lower extremities is shown in Figure 2A,B before topical treatment. As there was an incomplete resolution of the lesions, the patient was started on pimecrolimus, a calcineurin inhibitor topical cream, and the rash resolved after several months of therapy (Figure 3A). Given the resolution with topical anti-inflammatories and immunomodulators, the final diagnosis was a hypertrophic lichen planus secondary to pembrolizumab. It was suspected the endophytic lesions could reflect a non-neoplastic squamous proliferation, such as a pseudoepitheliomatous eruption of hypertrophic lichen planus, whereas the other possibility was eruptive keratoacanthomas. The patient has been able to continue taking pembrolizumab with no evidence of recurrence of his prior cutaneous reactions (Figure 3B).

### 2.2. Case 2

This is an 84-year-old female who was initially diagnosed with acral lentiginous melanoma, BRAF mutation negative, 0.9 mm depth, Clark level 4, non-ulcerative, with no initial lymph node involvement. The melanoma was excised, and the patient was followed with surveillance imaging. After 4 years, she was found to have bilateral pulmonary and hepatic metastases that were biopsy-confirmed as recurrent melanoma. Subsequently, she was initiated on pembrolizumab as the first-line treatment for her metastatic melanoma.

**Figure 4:**
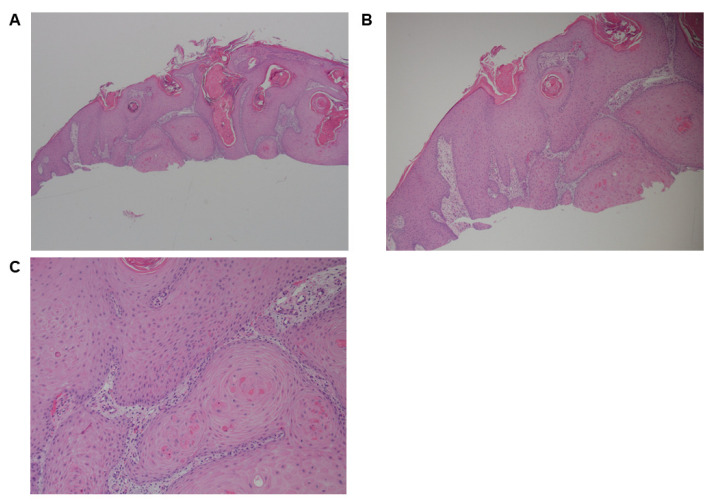
Case 2. Initial skin biopsies after pembrolizumab initiation. (**A**,**B**) Leg shave biopsy was interpreted as squamous cell carcinoma. Biopsy shows an endophytic squamous cell proliferation extending to the deep margin. The adjacent epidermis is acanthotic but not dysplastic. (**C**) The endophytic squamous proliferation shows no cytologic atypia.

**Figure 5:**
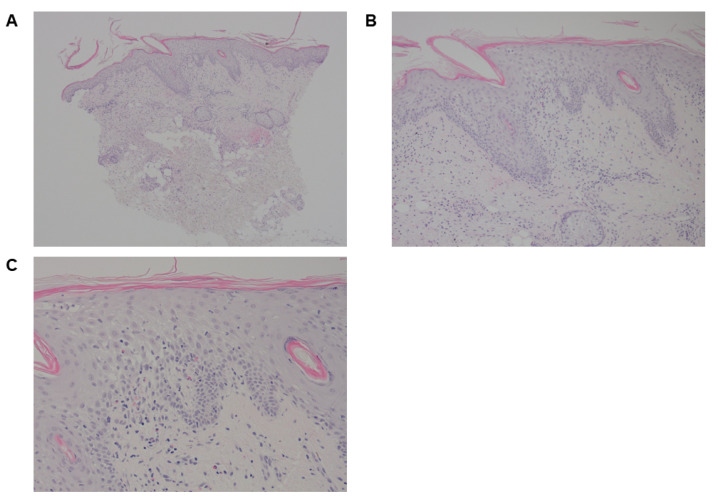
Case 2. (**A**) Skin punch biopsy of the erythrodermic reaction. (**B**,**C**) Show the superficial perivascular inflammatory dermatitis which included lymphocytes, histiocytes, plasma cells, and scattered eosinophils.

After 2 months of ICI therapy, the patient developed pruritic keratinous lesions on the lower limbs and was prescribed a betamethasone valerate cream, which had no effect. The lesions were then biopsied and interpreted as squamous cell carcinoma (Figure 4A–C). As such, they were removed via liquid nitrogen and curettage. The lesions unfortunately continued to recur and progressed in a more proximal distribution. Repeat biopsies again demonstrated squamous cell carcinoma. A prescription of 5-fluorouracil cream was prescribed but was ineffective and eventually discontinued. The lower limb lesions continued to progress, appearing on the proximal trunk, arms and back with blistering, ulcerations, and significant erythema 11 months after the 1st lesion’s appearance. The patient was admitted to the hospital due to the progressive nature of these lesions and was found to have oral mucous membrane involvement. During her admission, she developed a full-body erythrodermic reaction. Repeat biopsies were undertaken and they showed dermatitic changes with infiltration of lymphohistiocytic cells, plasmacytic cells, eosinophils, and neutrophils (Figure 5A–C). She was diagnosed with immune-checkpoint induced blistering dermatitis. She was initiated on 1 mg/kg of prednisone and steroid cream with significant improvement in the symptoms and regression of her lesions. She had intermitted flares while being tapered on prednisone and was left on 5 mg of prednisone for maintenance.

Five months after tapering the prednisone, she developed new verrucous and papillomatosis growths in the lower limbs which were biopsied and interpreted as squamous cell carcinoma. These ulcerating and violaceous plaque lesions were initially treated with 5-fluorouracil cream. A biopsy of a new lesion in the back showed non-specific chronic inflammatory infiltrates. The patient eventually had a biopsy repeat of the lower limb which showed inflamed and edematous highly vascular connective tissue in which there was a mixed infiltrate of lymphocytes, histiocytes, neutrophils, and plasma cells. No evidence of malignancy was seen. The patient was restarted on oral steroids with a gradual year-long taper, and the lesions gradually resolved and did not recur. Her melanoma remained stable for nearly 3 years without intercurrent anti-cancer therapy.

## 3. Discussion

Cutaneous lichenoid reactions are thought to be an autoimmune reaction to a self-antigen that leads to T-cell recruitment and immune activation at the dermal–epidermal junction [16]. Consequently, immune checkpoint inhibition may unmask an underlying self-reactive T-cell population. There is sparse literature reporting the effect of CTLA-4 inhibition alone inducing a lichenoid reaction and the reported literature mainly involves PD-1/PD-L1 blockade. In a small case series of patients referred to a dermatology clinic for cutaneous irAEs, 94% of biopsied patients had evidence of lichenoid dermatitis [16]. Lichenoid dermatitis can be triggered due to environmental factors; one common etiology is medication-induced [17]. For patients who were previously exposed to medications associated with lichenoid reactions before ICI use, it has been postulated that PD-1/PD-L1 antagonism may unmask a pre-existing immune response to one of these antigens that were previously suppressed through immune self-tolerance [16]. A similar mechanism has also been proposed in the pathogenesis of ICI-associated acute interstitial nephritis (AIN) [18]. An example of this mechanism is a case report of a patient who previously had controlled lichen planus which flared after nivolumab initiation [19]. In addition, another case reported infiltration of CD8+ PD-1+ T cells in the dermis for a patient with a pembrolizumab-induced skin rash which implicates the direct role of PD-1 and T-cell effector response in the skin [20].

The American Society of Clinical Oncology (ASCO), the European Society for Medical Oncology (ESMO), and the Society for Immunotherapy of Cancer (SITC) published guidelines for the management of cutaneous adverse events. The most severe dermatologic reactions require an urgent referral and include those which are blistering or bullous, involve mucocutaneous sites, or are associated with significant pain as there could be a rapid progression of an evolving SCAR. All guidelines recommend topical glucocorticoids and topical emollients for the treatment of grade 1–2 rashes. Topical therapies that can be considered include topical steroids or topical calcineurin inhibitors (TCI), such as pimecrolimus or tacrolimus. The appropriate selection of a topical agent will depend on the severity of the presentation and the location of the rash. Patients presenting with severe eruptions on the body, extremities, and scalp can be started on a potent topical steroid such as 0.05% clobetasol twice a day as needed for 6 weeks, then reassessed. If responding well, the patient can be switched to a TCI (e.g., 0.1% tacrolimus) or a less potent topical steroid such as 0.05% desonide or 1 % hydrocortisone to minimize potential adverse events. Topical steroids may induce dermatitis, atrophy, pruritus, and telangiectasia. Prolonged use of potent topical steroids is a risk factor for adrenal insufficiency. TCIs may induce local erythema, burning or stinging sensation, and pruritus.

Skin eruptions on the face, skin folds, or genitals should be treated with less potent steroids such as a desonide cream or TCIs applied twice a day as needed and reassessed at 6 weeks. The prescribing physician should consider the vehicle of the topical agent to improve compliance. For example, ointments or creams are reasonable to start for non-hairy skin. Lotions, gels, or solutions can be used for hairy areas. Skin folds can be treated with creams or lotions [21]. Specific formulations for each steroid cream will depend on local availability and some examples are referenced accordingly [22].

If there is a suspicion of grade 2 skin reactions that are refractory to topical treatment, ASCO recommends prednisone at 1 mg/kg/day while ESMO suggests a lower range of 0.5–1 mg/kg/day [23,24]. Adjunctive therapies for pruritis include antihistamine therapy and for refractory cases, gabapentinoids can be considered if there is pruritis with no rash [25]. For grades 3 and above, each guideline recommends cessation of ICI therapy, consideration of skin biopsy, and involvement of dermatology. Notable high-risk features, such as the red flag features described above, will warrant immediate therapy cessation, additional workup, and urgent dermatology involvement along with additional specialty involvement depending on which systems are involved.

Further investigations can include salt-split skin technique biopsy, direct immunofluorescence, and serological testing for immunobullous diseases. This may help differentiate unclear initial presentations, for example, a case of lichen planus due to ICI therapy initially presenting as bullous vesicular lesions with oral mucosal involvement has been reported in the literature [26]. Notably, more recent literature suggests that lichenoid reactions can progress to higher grades with possible fatal complications [27,28]. Steroid-sparing agents should be strongly considered for patients if the rash is persistent or prolonged steroid use is anticipated and should be undertaken in consultation with a dermatologist. However, most lichenoid reactions described in the literature were mild and almost 80% of lesions were controlled with topical therapies such as corticosteroids, fluorouracil, or tacrolimus [29]. Narrowband UV-B phototherapy and oral acitretin use have also been described in several case reports [29,30]. For severe lichenoid eruptions, SITC suggests the consideration of infliximab [25].

There is no clear data or guidance on which patient populations should be re-challenged. In grade 1–2 rashes, there may be a consideration depending on the refractory nature of the clinical course and input from a multidisciplinary perspective. It is not recommended to re-challenge grade 3 and above reactions. Notably, skin irAEs have been associated with improved disease control and treatment response possibly owing to robust activation of the immune system [31,32]. In one study, patients who developed eczema, lichenoid reaction, or vitiligo (reactions with suspected immune origin in the etiology) had improved progression-free survival compared to those who did not [33]. Our patients, since initiating treatment, have had prolonged disease control. In addition, both patients developed other irAEs, such as thyroiditis and type 1 diabetes, which in combination with skin irAEs (i.e., multisystem irAEs) have been associated with improved overall survival which is either a representation of a profoundly activated immune system or may represent common autoantigens shared between multiple irAEs [34].

There are case reports in the literature describing similar scenarios of lichen planus misdiagnosed as squamous cell carcinoma after skin biopsy as there are overlapping clinical, and histopathological features between the two entities [12,13,14,15]. Similar to other case reports, lesions appeared after several months of PD-1/PD-L1 blockade for our patients [14,35,36]. Pruritic lichenoid planus drug-induced rashes may result in florid squamous endophytic proliferation due to rubbing. The pseudoepitheliomatous squamous proliferation may emulate a kerato-acanthomatous well-differentiated squamous cell carcinoma as in case 1. Knowledge of a pruritic rash following ICI therapy and sampling of the adjacent non-nodular areas is important for revealing the underlying lichenoid inflammatory nature [37]. In our cases, the distinguishing factors that favoured a non-neoplastic process included a lack of evidence for ICIs in the oncogenesis of squamous cell carcinoma, the appearance of the lesions as hyperkeratotic, and the appearance of multiple lesions at the same time in the context of immunotherapy. Biopsy features that made squamous cell carcinoma less likely were lichenoid inflammatory changes, absence of dysplasia, and absence of invasion into the dermis.

In general, clinicians should be aware of the possibility that the appearance of multiple simultaneous violaceous rashes for a patient on PD-1/PD-L1 inhibitor therapy should prompt suspicion of an autoinflammatory skin reaction even if the histopathology may suggest squamous cell carcinoma. As described in the literature and our cases, this can be a common misdiagnosis and an expert dermatopathology re-evaluation is warranted. A trial of oral steroids may be warranted if lesions continue to progress. Confirming the diagnosis of a cutaneous irAE, as opposed to an SCC, has several management implications, such as avoidance of invasive interventions, the decision to discontinue ICI if severe toxicities are recognized, and prompt initiation of immunosuppressive therapy. The spectrum of cutaneous irAE remains to be fully characterized and this case report highlights the importance of a greater awareness of the possibility of autoinflammatory cutaneous reaction for patients on PD-1/PD-L1 inhibitor therapy despite conflicting histopathological results.

## Data Availability

Data sharing not applicable.
